# Supplementary material for: Targets of drugs are generally, and targets of drugs having side effects are specifically good spreaders of human interactome perturbations
Source: Sci Rep. 2015 May 11;5:10182. doi: 10.1038/srep10182 (PMC4426692; doi:10.1038/srep10182)
Supplement: Supplementary Information [file srep10182-s1.pdf]

Supplementary Information

Targets of drugs are generally, and targets of drugs having side effects are specifically good spreaders of human interactome perturbations

Áron R. Perez-Lopez[1,*], Kristóf Z. Szalay[1], Dénes Türei[2,+], Dezső Módos[2,3], Katalin Lenti[3], Tamás Korcsmáros[2,4,5] and Peter Csermely[1,#]

1 Department of Medical Chemistry, Semmelweis University, P.O. Box 260, H-1444 Budapest 8, Hungary; **2** Department of Genetics, Eötvös Loránd University, Pázmány P. s. 1C, H-1117 Budapest, Hungary; **3** Department of Morphology and Physiology, Faculty of Health Sciences, Semmelweis University, Vas u. 17, H-1088 Budapest, Hungary; **4** TGAC, The Genome Analysis Centre, Norwich, UK; **5** Gut Health and Food Safety Programme, Institute of Food Research, Norwich, UK

**Áron R. Perez Lopez is a high school student of the Apáczai Csere János High School of the Eötvös Loránd University, Papnövelde u. 4-6, H-1053 Budapest, Hungary; +current address: European Bioinformatics Institute (EMBL-EBI), Wellcome Trust Genome Campus, Cambridge CB10 1SD, UK*
#Corresponding author, E-mail: csermely.peter@med.semmelweis-univ.hu

Table of Contents

Supplementary Figures **3**
Figure 1 | Cumulative silencing time distribution of drug targets and non-target proteins with a starting energy of 10,000 and a dissipation value of 5 3
Figure 2 | Cumulative silencing time distribution of drug targets and non-target proteins with a starting energy of 10,000 and a dissipation value of 1 4
Figure 3 | Cumulative silencing time distribution of drugs and non-target proteins with starting energy of 1,000 and a dissipation value of 5 with distributed starting energy among multiple targets 5
Figure 4 | Cumulative silencing time distribution of drug target proteins and non-target proteins with a starting energy of 1000 and a dissipation value of 5 using a 50% smaller interactome 6
Figure 5 | Cumulative perturbation reach distribution of drug targets and non-target proteins with a starting energy of 10,000 and a dissipation value of 5 7
Figure 6 | Cumulative perturbation reach distribution of drug targets and non-target proteins with a starting energy of 10,000 and a dissipation value of 1 8
Figure 7 | Cumulative perturbation reach distribution of drugs and non-target proteins with starting energy of 10,000 and a dissipation value of 1 with distributed starting energy among multiple targets 9
Figure 8 | Cumulative silencing time distribution of targets of drugs used in the treatment of colorectal cancer and type 2 diabetes mellitus 10
Figure 9 | Human interactome distance between drug targets used in the treatment of colorectal cancer and type 2 diabetes, between proteins related to these diseases and randomly selected proteins 11

Figure 10 | Human protein-protein interaction network of the proteins related to colorectal cancer and type 2 diabetes and the drug targets used in the treatment of these diseases 13
Supplementary Tables 15
Table 1 | Drugs obtained from the DrugBank database, which have known side effects in the SIDER database 15
Table 2 | The keywords used in the filtering of the DrugBank database and their occurrences 21
Table 3 | Drugs obtained from the DrugBank database, which are used in the treatment of colorectal cancer and have no reported side effects in the SIDER database and their target proteins 22
Table 4 | Drugs obtained from the DrugBank database, which are used in the treatment of colorectal cancer and have known side effects in the SIDER database and their target proteins 23
Table 5 | Drugs obtained from the DrugBank database, which are used in the treatment of type 2 diabetes and have no reported side effects in the SIDER database and their target proteins 24
Table 6 | Drugs obtained from the DrugBank database, which are used in the treatment of type 2 diabetes and have known side effects in the SIDER database and their target proteins 25
Table 7 | Mutated genes in colorectal cancer and their corresponding proteins 26
Table 8 | Mutated genes in type 2 diabetes and their corresponding proteins 27
Table 9 | Average human interactome centralities of target proteins of drugs against colorectal cancer and type 2 diabetes 28
Table 10 | Average network distance between drug targets without known side effects used in the treatment of colorectal cancer and colorectal cancer-associated proteins 29
Table 11 | Average network distance between drug targets with known side effects used in the treatment of colorectal cancer and colorectal cancer-associated proteins 30
Table 12 | Average network distance between drug targets without known side effects used in the treatment of type 2 diabetes and diabetes-associated proteins 31
Table 13 | Average network distance between drug targets with known side effects used in the treatment of type 2 diabetes and diabetes-associated proteins 32
Supplementary References 33

Supplementary Figures

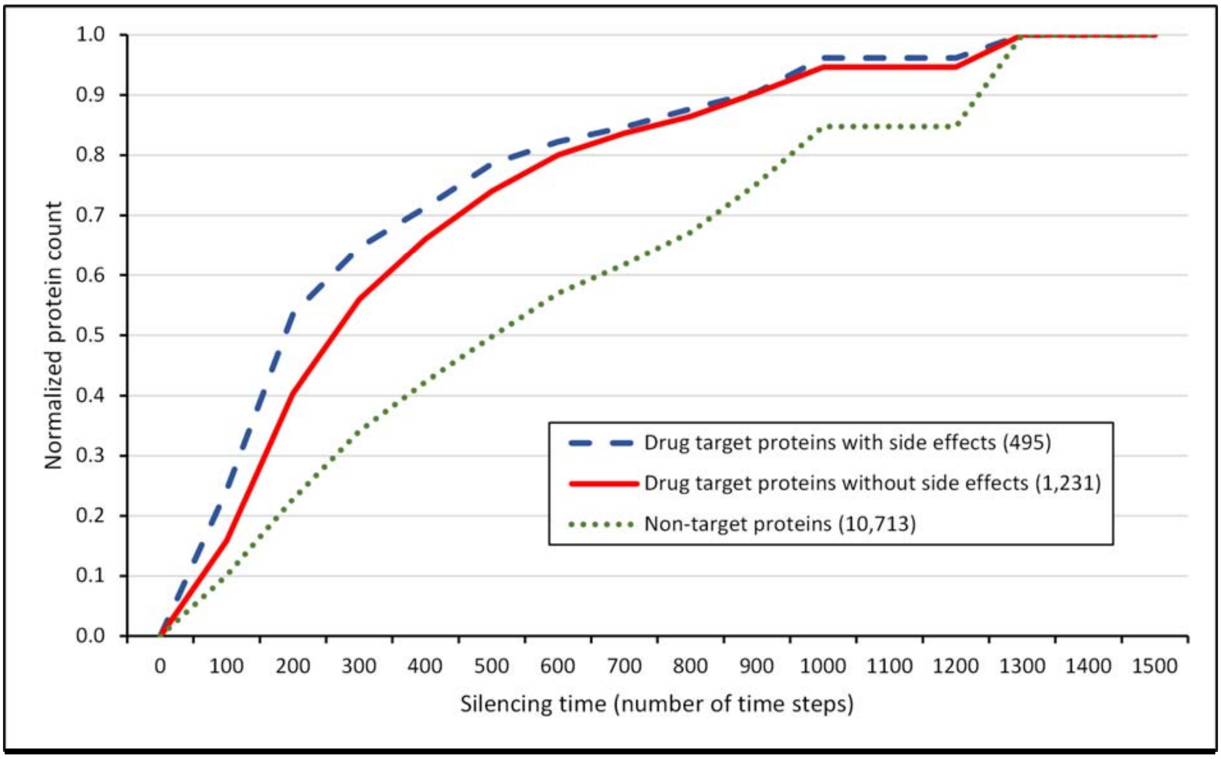

Figure 1 | Cumulative silencing time distribution of drug targets and non-target proteins with a starting energy of 10,000 and a dissipation value of 5. The diagram shows the cumulative distribution of the normalized number of proteins with given silencing times, which are drug targets with known side effects (blue dashed line), which are drug targets without known side effects (red solid line) and which are not drug targets (green dotted line). The number of proteins was normalized by dividing the number of proteins in each silencing time range by the total number of proteins allowing a better comparison. The total number of drug targets with and without side effects, and non-target proteins was 495, 1,231 and 10,713, respectively. The figure shows the 99.99% of all proteins (having a silencing time below 1500). The human interactome containing 12,439 proteins and 174,666 edges was built from the STRING database[1], 1,726 human drug targets were obtained from the DrugBank database[2] and 99,423 drug-side effect pairs were taken from the SIDER database[3]. Silencing times were calculated separately for every protein with the Turbine program[4] as described in the Methods section of the main text with a starting energy of 10,000 and a dissipation value of 5 units. Statistical analysis was performed using the Mann-Whitney (Wilcoxon rank sum) test function of the R package[5]. There was a statistically significant difference (p=1.701e-5) between the silencing times of drug targets with known side effects and the silencing times of drug targets without known side effects. The difference between the silencing times of drug targets and non-target proteins was also statistically significant (p=2.2e-16).

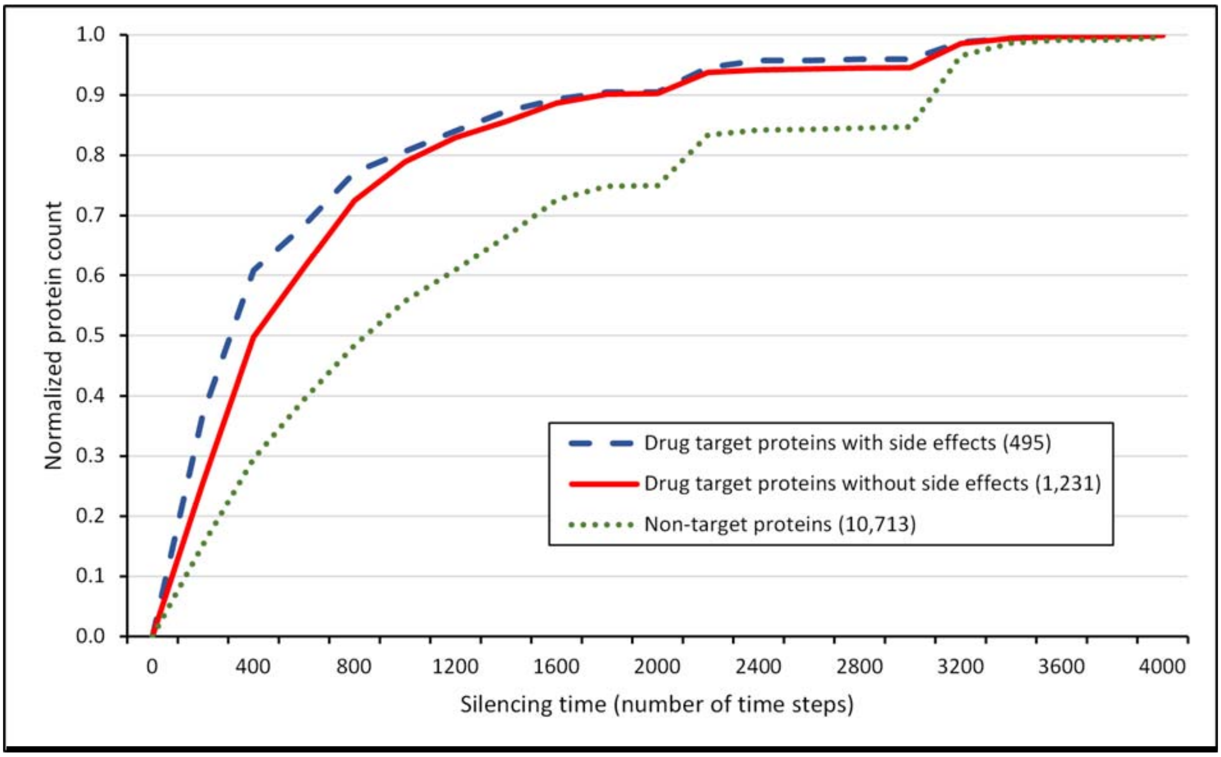

Figure 2 | Cumulative silencing time distribution of drug targets and non-target proteins with a starting energy of 10,000 and a dissipation value of 1. The diagram shows the cumulative distribution of the normalized number of proteins with given silencing times, which are drug targets with known side effects (blue dashed line), which are drug targets without known side effects (red solid line) and which are not drug targets (green dotted line). The number of proteins was normalized by dividing the number of proteins in each silencing time range by the total number of proteins allowing a better comparison. The total number of drug targets with and without side effects, and non-target proteins was 495, 1,231 and 10,713, respectively. The figure shows 99.61% of all proteins (having a silencing time below 4000). The human interactome containing 12,439 proteins and 174,666 edges was built from the STRING database[1], 1,726 human drug targets were obtained from the DrugBank database[2] and 99,423 drug-side effect pairs were taken from the SIDER database[3]. Silencing times were calculated separately for every protein with the Turbine program[4] as described in the Methods section of the main text with a starting energy of 10,000 and a dissipation value of 1 unit. Statistical analysis was performed using the Mann-Whitney (Wilcoxon rank sum) test function of the R package[5]. There was a statistically significant difference (p=9.635e-6) between the silencing times of drug targets with known side effects and the silencing times of drug targets without known side effects. The difference between the silencing times of drug targets and non-target proteins was also statistically significant (p=2.2e-16).

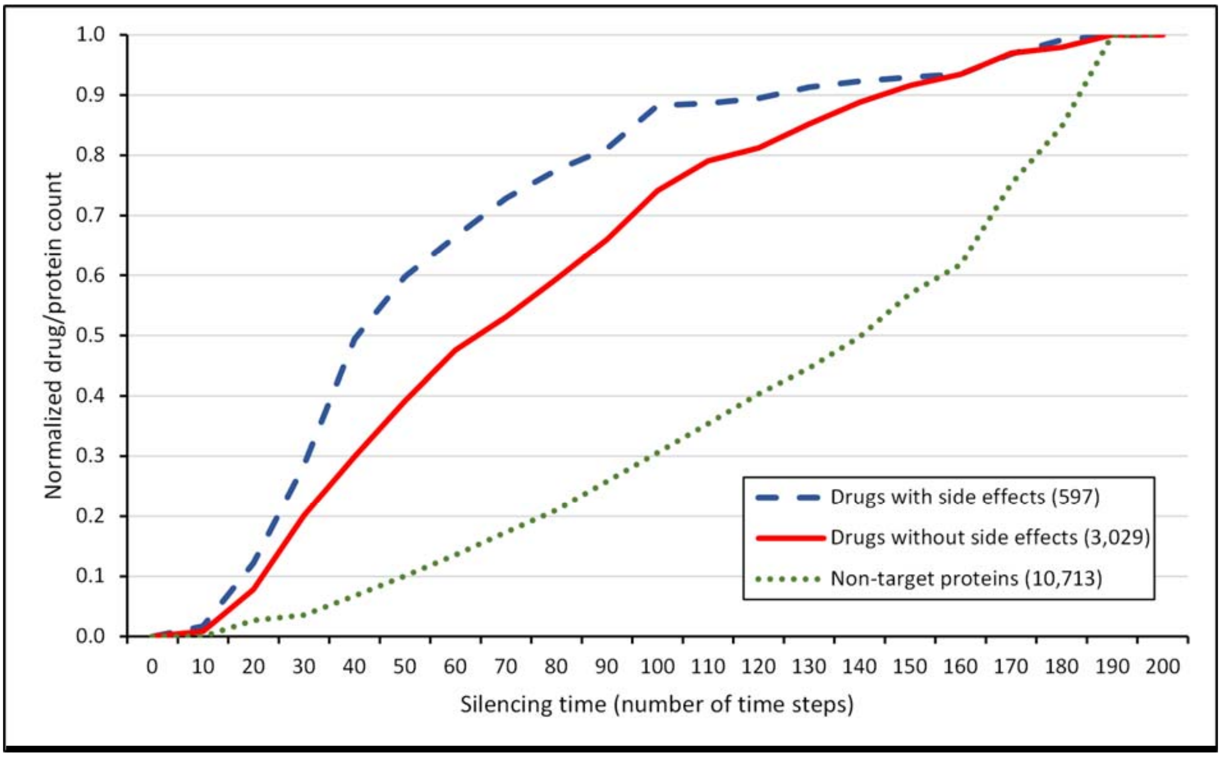

Figure 3 | Cumulative silencing time distribution of drugs and non-target proteins with starting energy of 1,000 and a dissipation value of 5 with distributed starting energy among multiple targets. The diagram shows the cumulative silencing time distribution of the normalized number of drugs with known side effects (blue dashed line), drugs without known side effects (red solid line) and non-target proteins (green dotted line). The number of proteins/drugs was normalized by dividing the number of proteins/drugs in each silencing time range by the total number of proteins/drugs allowing a better comparison. The total number of drugs with and without side effects, and non-target proteins was 597, 3,029 and 10,713, respectively. The human interactome containing 12,439 proteins and 174,666 edges was built from the STRING database[1], 3,626 human drugs were obtained from the DrugBank database[2] and 99,423 drug-side effect pairs were taken from the SIDER database[3]. Silencing times were calculated separately for every protein/drug with the Turbine program[4] as described in the Methods section of the main text with a starting energy of 1000 and a dissipation value of 5 units. In case of drugs with multiple targets, the starting energy was distributed evenly among the drug targets. Statistical analysis was performed using the Mann-Whitney (Wilcoxon rank sum) test function of the R package[5]. There was a statistically significant difference (p=2.2e-16) between the silencing times of drugs with known side effects and the silencing times of drugs without known side effects. The difference between the silencing times of drugs and non-target proteins was also statistically significant (p=2.2e-16).

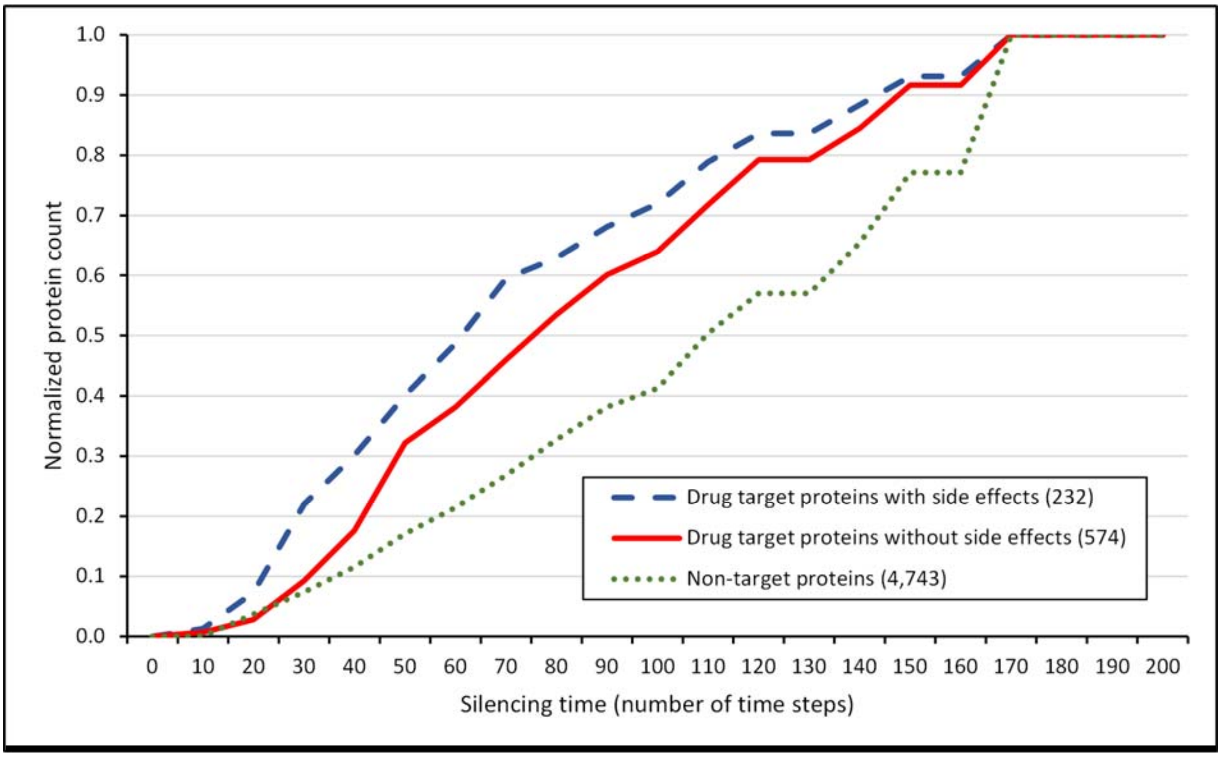

Figure 4 | Cumulative silencing time distribution of drug target proteins and non-target proteins with a starting energy of 1000 and a dissipation value of 5 using a 50% smaller interactome. The diagram shows the cumulative distribution of the normalized number of proteins with given silencing times, which are drug targets with known side effects (blue dashed line), which are drug targets without known side effects (red solid line) and which are not drug targets (green dotted line). The number of proteins was normalized by dividing the number of proteins in each silencing time range by the total number of proteins allowing a better comparison. The total number of drug targets with and without side effects, and non-target proteins was 495, 1,231 and 10,713, respectively. The human interactome containing 12,439 proteins and 174,666 edges was built from the STRING database[1], 1,726 human drug targets were obtained from the DrugBank database[2] and 99,423 drug-side effect pairs were taken from the SIDER database[3]. 50% of the original interactome proteins were deleted randomly. The giant component of the remaining interactome contained 5,549 proteins (45%), 806 drug target proteins total (47%) and 232 drug targets with known side effects (47%). Silencing times were calculated separately for every protein with the Turbine program[4] as described in the Methods section of the main text with a starting energy of 1,000 and a dissipation value of 5 units. Statistical analysis was performed using the Mann-Whitney (Wilcoxon rank sum) test function of the R package[5]. There was a statistically significant difference (p=3.368e-4) between the silencing times of drug targets with known side effects and the silencing times of drug targets without known side effects. The difference between the silencing times of drug targets and non-target proteins was also statistically significant (p=2.2e-16).

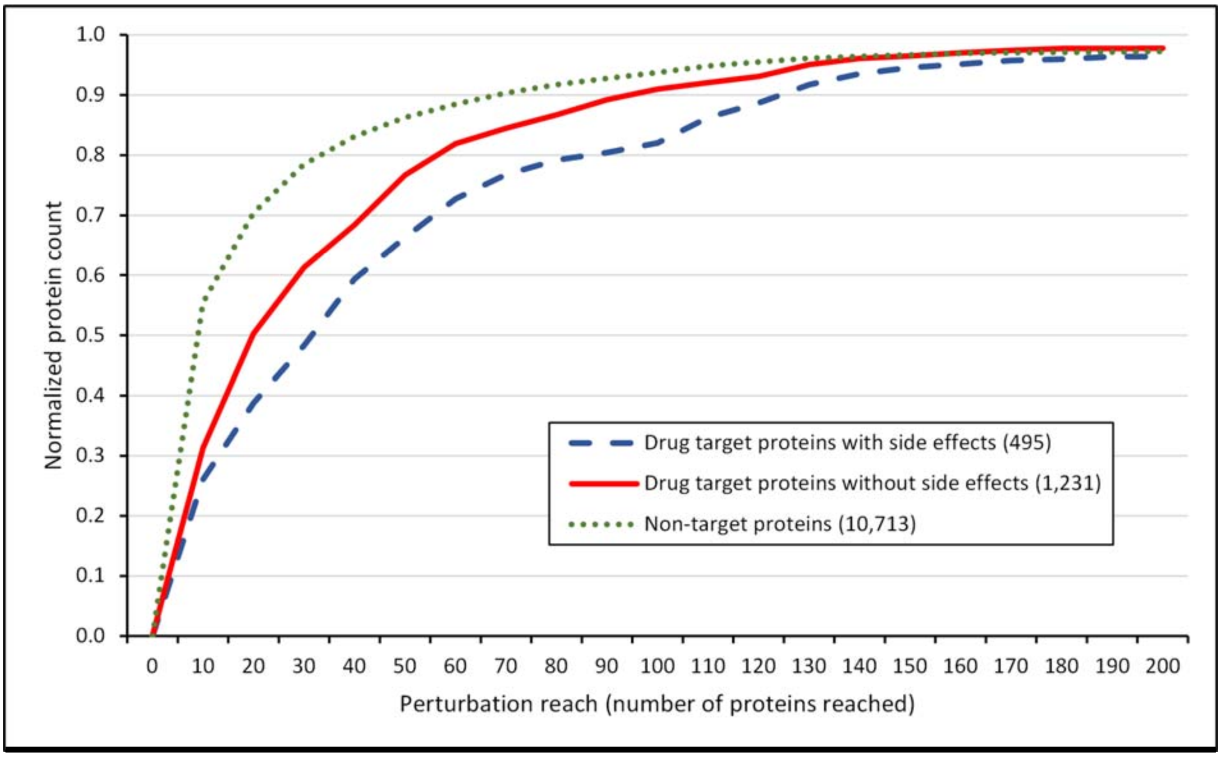

Figure 5│Cumulative perturbation reach distribution of drug targets and non-target proteins with a starting energy of 10,000 and a dissipation value of 5. The diagram shows the cumulative distribution of the normalized number of proteins with given perturbation reach values, which are drug targets with known side effects (blue dashed line), which are drug targets without known side effects (red solid line) and which are not drug targets (green dotted line). The number of proteins was normalized by dividing the number of proteins in each perturbation reach range by the total number of proteins allowing a better comparison. The total number of drug targets with and without side effects, and non-target proteins was 495, 1,231 and 10,713, respectively. The figure shows 97.25% of all proteins (having a perturbation reach below 200 proteins reached). The human interactome containing 12,439 proteins and 174,666 edges was built from the STRING database[1], 1,726 human drug targets were obtained from the DrugBank database[2] and 99,423 drug-side effect pairs were taken from the SIDER database[3]. Perturbation reach values were calculated separately for every protein with the Turbine program[4] as described in the Methods section of the main text with a starting energy of 10,000 and a dissipation value of 5 units. Statistical analysis was performed using the Mann-Whitney (Wilcoxon rank sum) test function of the R package[5]. There was a statistically significant difference (p=1.663e-5) between the perturbation reach values of drug targets with known side effects and the perturbation reach values of drug targets without known side effects. The difference between the perturbation reach values of drug targets and non-target proteins was also statistically significant (p=2.2e-16).

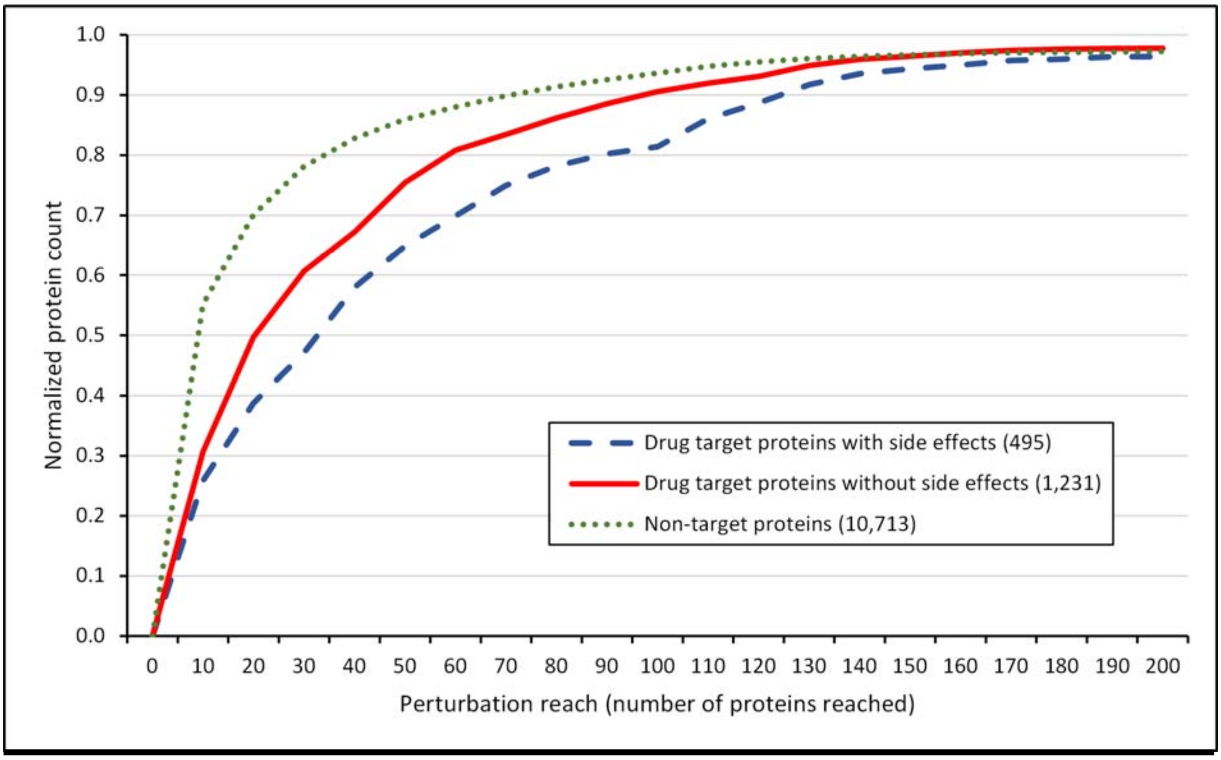

Figure 6│Cumulative perturbation reach distribution of drug targets and non-target proteins with a starting energy of 10,000 and a dissipation value of 1. The diagram shows the cumulative distribution of the normalized number of proteins with given perturbation reach values, which are drug targets with known side effects (blue dashed line), which are drug targets without known side effects (red solid line) and which are not drug targets (green dotted line). The number of proteins was normalized by dividing the number of proteins in each perturbation reach range by the total number of proteins allowing a better comparison. The total number of drug targets with and without side effects, and non-target proteins was 495, 1,231 and 10,713, respectively. The figure shows 97.25% of all proteins (having a perturbation reach below 200 proteins reached). The human interactome containing 12,439 proteins and 174,666 edges was built from the STRING database[1], 1,726 human drug targets were obtained from the DrugBank database[2] and 99,423 drug-side effect pairs were taken from the SIDER database[3]. Perturbation reach values were calculated separately for every protein with the Turbine program[4] as described in the Methods section of the main text with a starting energy of 10,000 and a dissipation value of 1 unit. Statistical analysis was performed using the Mann-Whitney (Wilcoxon rank sum) test function of the R package[5]. There was a statistically significant difference (p=1.49e-5) between the perturbation reach values of drug targets with known side effects and the perturbation reach values of drug targets without known side effects. The difference between the perturbation reach values of drug targets and non-target proteins was also statistically significant (p=2.2e-16).

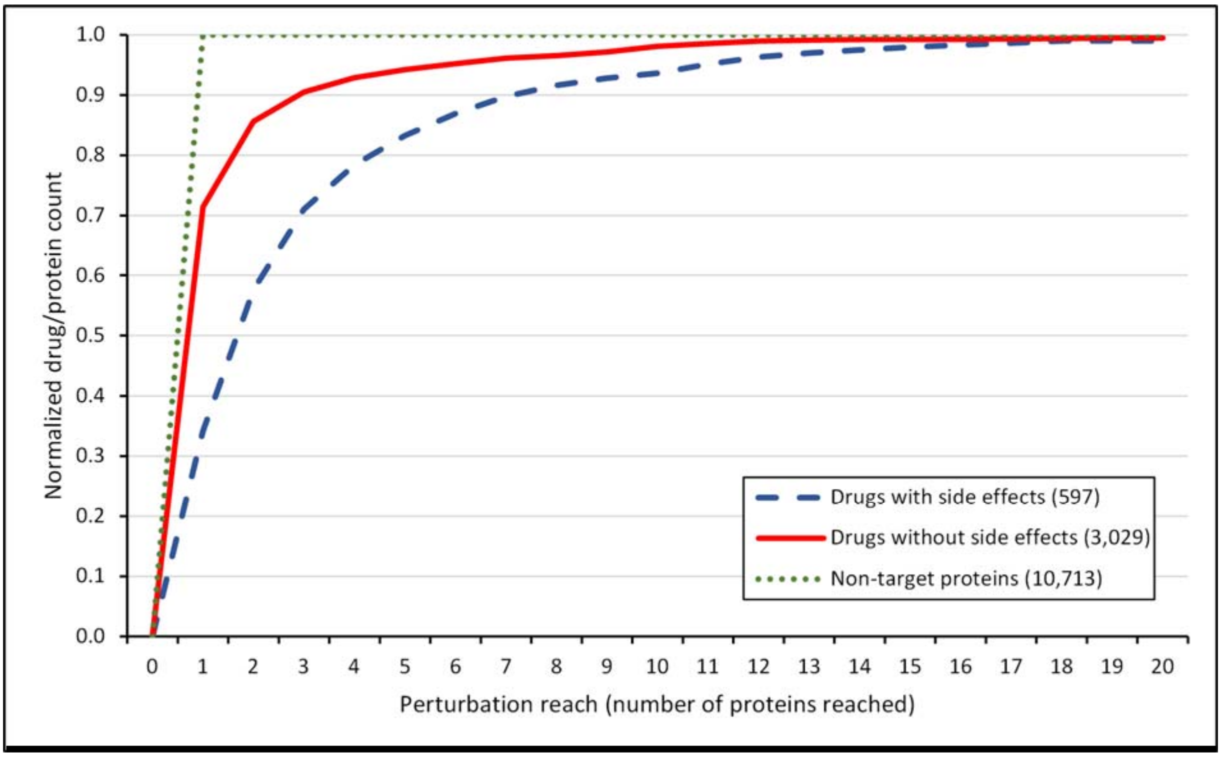

Figure 7 | Cumulative perturbation reach distribution of drugs and non-target proteins with starting energy of 10,000 and a dissipation value of 1 with distributed starting energy among multiple targets. The diagram shows the cumulative perturbation reach distribution of the normalized number of drugs with known side effects (blue dashed line), drugs without known side effects (red solid line) and non-target proteins (green dotted line). The number of proteins/drugs was normalized by dividing the number of proteins/drugs in each perturbation reach range by the total number of proteins/drugs allowing a better comparison. The total number of drugs with and without side effects, and non-target proteins was 597, 3,029 and 10,713, respectively. The figure shows 99.58% of all proteins/drugs (having a perturbation reach below 400 proteins reached). The human interactome containing 12,439 proteins and 174,666 edges was built from the STRING database[1], 3,626 human drugs were obtained from the DrugBank database[2] and 99,423 drug-side effect pairs were taken from the SIDER database[3]. Perturbation reach values were calculated separately for every protein/drug with the Turbine program[4] as described in the Methods section of the main text with a starting energy of 10,000 and a dissipation value of 1 unit. In case of drugs with multiple targets, the starting energy was distributed evenly among the drug targets. Statistical analysis was performed using the Mann-Whitney (Wilcoxon) test function of the R package[5]. There was a statistically significant difference (p=6.176e-8) between the perturbation reach values of drugs with known side effects and the perturbation reach values of drugs without known side effects. The difference between the perturbation reach values of drugs and non-target proteins was also statistically significant (p=2.2e-16).

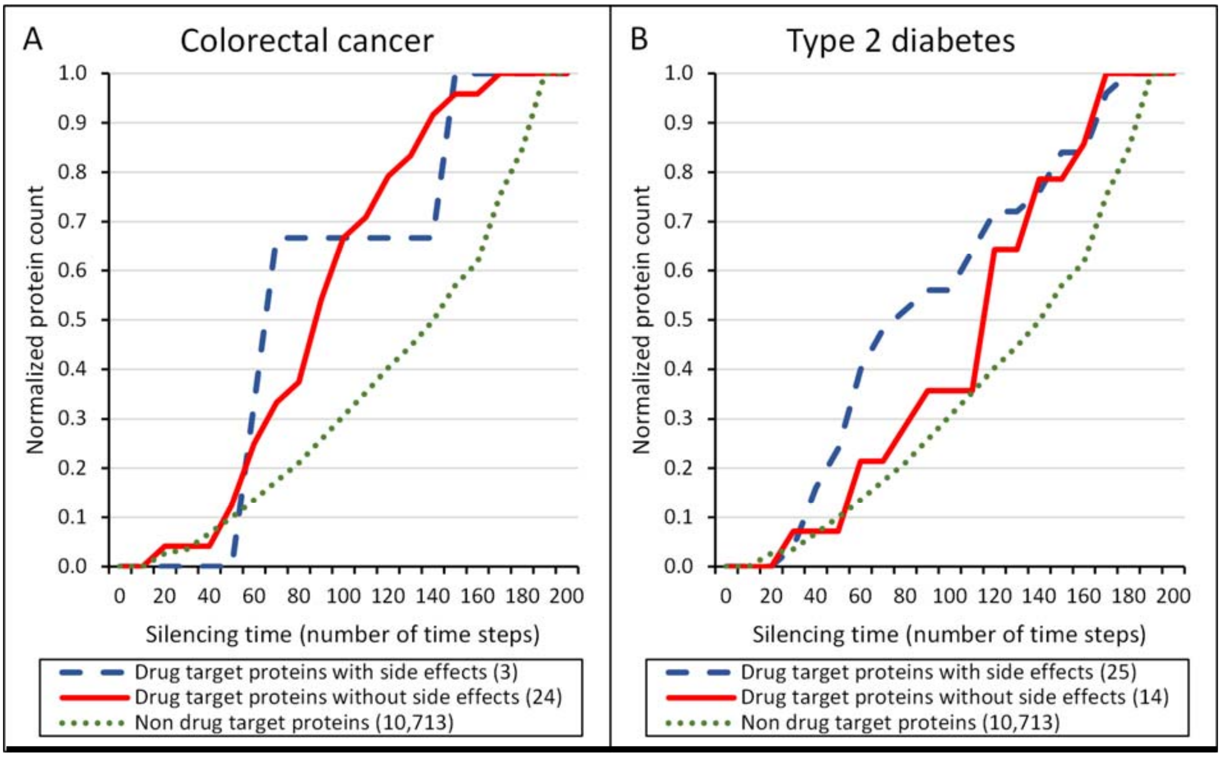

Figure 8 | Cumulative silencing time distribution of targets of drugs used in the treatment of colorectal cancer and type 2 diabetes mellitus. The diagram shows the cumulative distribution of the normalized number of proteins with given silencing times, which are drug targets used in the treatment of the disease with known side effects (blue dashed line), which are drug targets used in the treatment of the disease without known side effects (red solid line) and which are not drug targets (green dotted line); for colorectal cancer (Panel A) and type 2 diabetes (Panel B). The number of proteins was normalized by dividing the number of proteins in each silencing time range by the total number of proteins allowing a better comparison. The total number of drug targets used in the treatment of colorectal cancer with and without side effects was 3 and 24, respectively, while for type 2 diabetes the total number of drug targets was 25 and 14, respectively. The human interactome containing 12,439 proteins and 174,666 edges was built from the STRING database[1], 1,726 human drug targets were obtained from the DrugBank database[2] and 99,423 drug-side effect pairs were taken from the SIDER database[3]. Silencing times were calculated separately for every protein with the Turbine program[4] as described in the Methods section of the main text with a starting energy of 1,000 and a dissipation value of 5 units. Statistical analysis was performed using the Mann-Whitney-Wilcoxon test of the R package[5]. No statistically significant difference could be shown between silencing times of targets with known side effects and silencing times of targets without known side effects of drugs used in the treatment of colorectal cancer ($p=1$) and type 2 diabetes ($p=0.2593$). However, the difference between the silencing times of drug targets and non-target proteins was statistically significant for drug targets used in the treatment of both colorectal cancer ($p=3.367e-5$) and type 2 diabetes ($p=5.88e-5$).

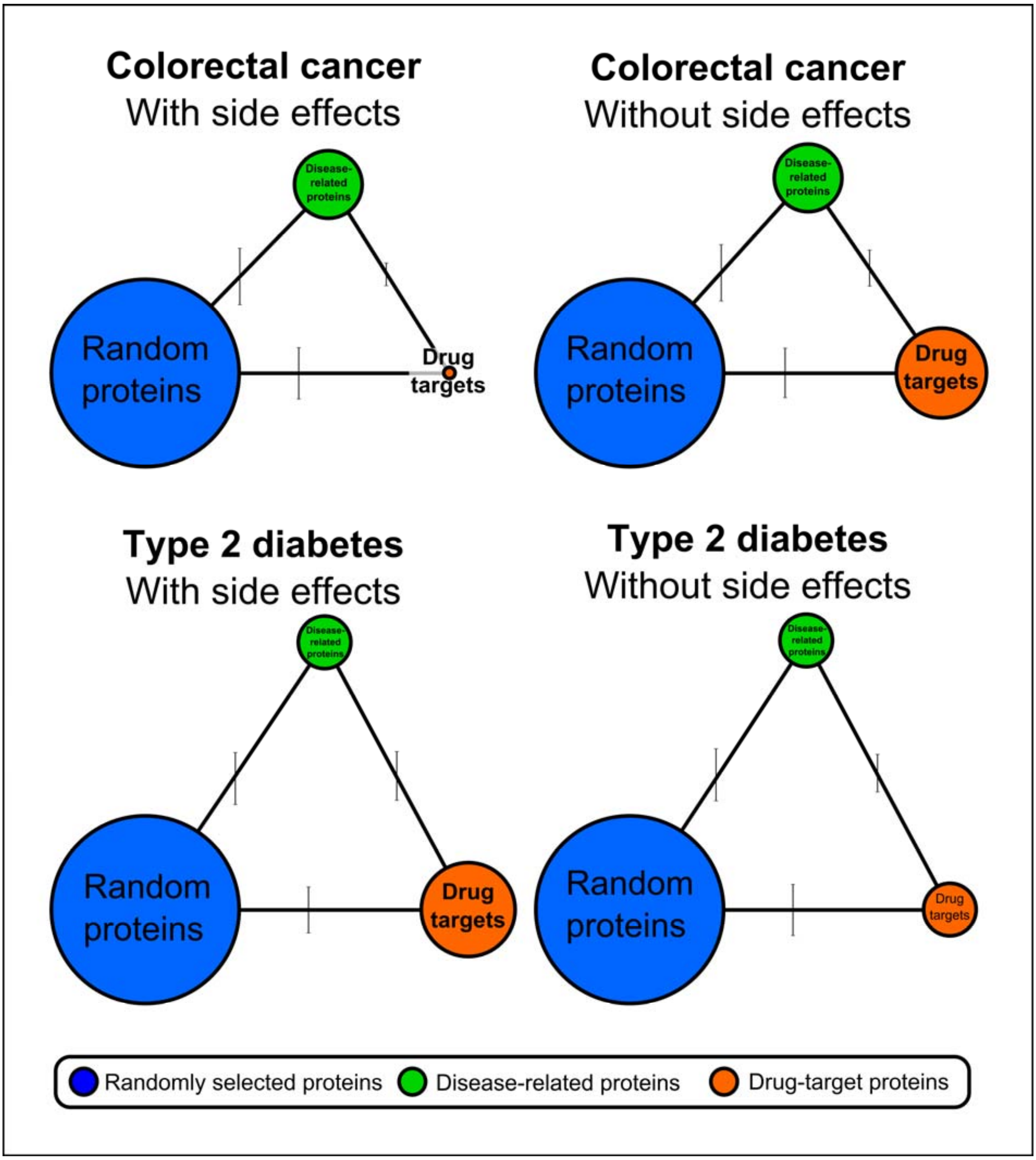

Figure 9 │ Human interactome distance between drug targets used in the treatment of colorectal cancer and type 2 diabetes, between proteins related to these diseases and randomly selected proteins. The figure shows the average human interactome distances between the following proteins: drug targets used in the treatment of colorectal cancer and type 2 diabetes with and without side effects (orange circles), proteins related to these diseases (green circles) and randomly selected proteins (blue circles). The sides of the triangles (the distance between the centres of the circles) are proportional to the average number of human interactome

edges between the respective protein groups, while the vertical lines associated with the sides of the triangles correspond to the standard deviation (SD). The average distance between randomly selected proteins and disease-related proteins was 2.82 edges (SD: 0.601) for colorectal cancer and 3.43 edges (SD: 0.557) for type 2 diabetes; between randomly selected proteins and drug targets with side effects was 3.24 edges (SD: 0.551) for colorectal cancer and 3.44 edges (SD: 0.490) for type 2 diabetes; between randomly selected proteins and drug targets without side effects was 3.32 edges (SD: 0.533) for colorectal cancer and 3.41 edges (SD: 0.545) for type 2 diabetes; between disease-related proteins and drug targets with side effects was 2.39 edges (SD: 0.242) for colorectal cancer and 3.23 edges (SD: 0.522) for type 2 diabetes; between disease-related proteins and drug targets without side effects was 2.53 edges (SD: 0.388) for colorectal cancer and 3.25 edges (SD: 0.402) for type 2 diabetes. Sizes of the circles are proportional to the number of proteins contained in each group. There were 50 randomly selected proteins; 18 colorectal cancer-related and 14 type 2 diabetes-related proteins; 3 drug targets with and 24 drug targets without side effects used in the treatment of colorectal cancer; 25 drug targets with and 14 drug targets without side effects used in the treatment of type 2 diabetes. The human interactome containing 12,439 proteins and 174,666 edges was built from the STRING database[1], 1,726 human drug targets were obtained from the DrugBank database[2] and 99,423 drug-side effect pairs were taken from the SIDER database[3]. Network distances were calculated as shortest paths using the Pajek programme[6] as described in the Methods section of the main text and are detailed in Tables 10-13. The figure was created using Inkscape[7].

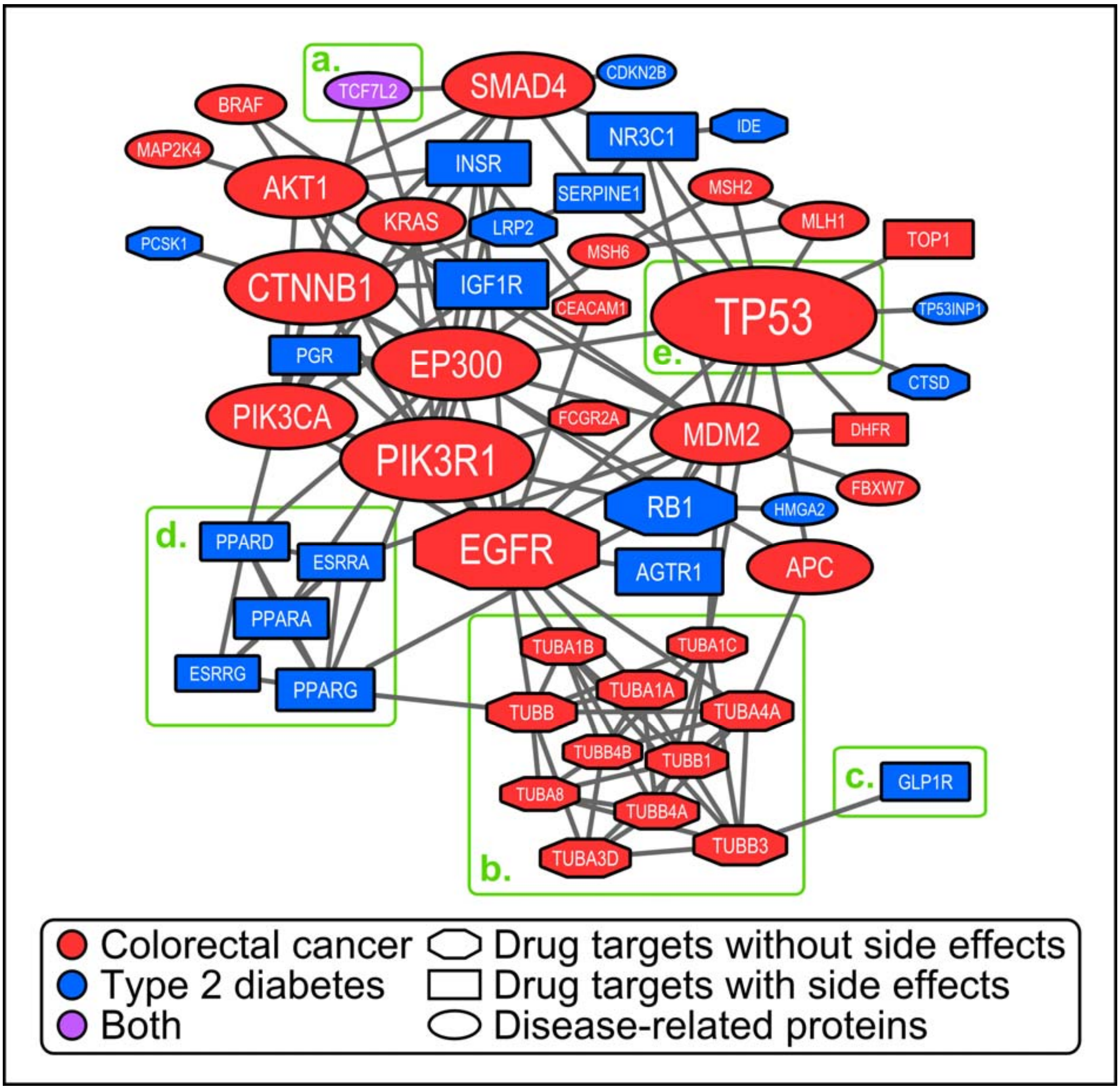

Figure 10│Human protein-protein interaction network of the proteins related to colorectal cancer and type 2 diabetes and the drug targets used in the treatment of these diseases. The figure shows the giant component of the human protein-protein interaction network containing the proteins related to colorectal cancer and type 2 diabetes mellitus and the drug targets used in the treatment of these diseases. Red nodes represent proteins or drug targets related to colorectal cancer, blue nodes represent those related to type 2 diabetes, while purple nodes represent those related to both. Ellipses, octagons and squares represent proteins related to diseases, drug targets without known side effects and drug targets with known side effects, respectively. Node highlighted by green box (a.) is the TCF7L2 protein related to both diseases, which is the transcription factor 7-like 2 participating in the Wnt signalling pathway and modulating MYC expression. The highly interconnected node cluster highlighted by green box (b.) contains 11 drug targets without known side effects used in the treatment of colorectal cancer, which are all

tubuline chain proteins. Node highlighted by green box (c.) representing protein GLP1R, the glucagon-like peptide 1 receptor, is connected only to node TUBB3 of the tubuline cluster (b.). The highly interconnected node cluster highlighted by green box (d.) contains 5 drug targets with known side effects used in the treatment of type 2 diabetes which are the peroxisome proliferator-activated receptors alpha (PPARA), gamma (PPARG) and delta (PPARD) and the estrogen-related receptors alpha (ESRRA) and gamma (ESSRG). The network hub highlighted by green box (e.) is TP53, the cellular tumour antigen p53. Node sizes are proportional to the degrees of the respective proteins in the full human protein-protein interaction network. All proteins here are referenced by their UniProt ID[9]. The human interactome containing 12,439 proteins and 174,666 edges was built from the STRING database[1], 1,726 human drug targets were obtained from the DrugBank database[2] and 99,423 drug-side effect pairs were taken from the SIDER database[3]. Node degrees were calculated with the Pajek programme[6] as described in the Methods section of the main text. The figure was created using Cytoscape[8] and Inkscape[7].

Supplementary Tables

Table 1 │ Drugs obtained from the DrugBank database, which have known side effects in the SIDER database

DBID	Drug Name
DB00001	Lepirudin
DB00006	Bivalirudin
DB00046	Insulin Lispro
DB00047	Insulin Glargine
DB00050	Cetrorelix
DB00063	Eptifibatide
DB00106	Abarelix
DB00115	Cyanocobalamin
DB00125	L-Arginine
DB00152	Thiamine
DB00162	Vitamin A
DB00175	Pravastatin
DB00176	Fluvoxamine
DB00177	Valsartan
DB00178	Ramipril
DB00180	Flunisolide
DB00182	Amphetamine
DB00184	Nicotine
DB00185	Cevimeline
DB00186	Lorazepam
DB00187	Esmolol
DB00188	Bortezomib
DB00191	Phentermine
DB00193	Tramadol
DB00195	Betaxolol
DB00197	Troglitazone
DB00198	Oseltamivir
DB00200	Hydroxocobalamin
DB00201	Caffeine
DB00202	Succinylcholine
DB00204	Dofetilide
DB00205	Pyrimethamine
DB00206	Reserpine
DB00208	Ticlopidine

DBID	Drug Name
DB00210	Adapalene
DB00211	Midodrine
DB00213	Pantoprazole
DB00214	Torasemide
DB00215	Citalopram
DB00216	Eletriptan
DB00218	Moxifloxacin
DB00222	Glimepiride
DB00227	Lovastatin
DB00228	Enflurane
DB00231	Temazepam
DB00240	Alclometasone
DB00242	Cladribine
DB00243	Ranolazine
DB00246	Ziprasidone
DB00247	Methysergide
DB00248	Cabergoline
DB00252	Phenytoin
DB00253	Medrysone
DB00257	Clotrimazole
DB00264	Metoprolol
DB00268	Ropinirole
DB00273	Topiramate
DB00276	Amsacrine
DB00277	Theophylline
DB00278	Argatroban
DB00280	Disopyramide
DB00281	Lidocaine
DB00282	Pamidronate
DB00284	Acarbose
DB00285	Venlafaxine
DB00286	Conjugated Estrogens
DB00287	Travoprost
DB00288	Amcinonide

DBID	Drug Name
DB00289	Atomoxetine
DB00292	Etomidate
DB00293	Raltitrexed
DB00295	Morphine
DB00296	Ropivacaine
DB00297	Bupivacaine
DB00302	Tranexamic Acid
DB00307	Bexarotene
DB00308	Ibutilide
DB00310	Chlorthalidone
DB00312	Pentobarbital
DB00313	Valproic Acid
DB00315	Zolmitriptan
DB00316	Acetaminophen
DB00317	Gefitinib
DB00318	Codeine
DB00320	Dihydroergotamine
DB00321	Amitriptyline
DB00323	Tolcapone
DB00324	Fluorometholone
DB00327	Hydromorphone
DB00328	Indomethacin
DB00331	Metformin
DB00332	Ipratropium bromide
DB00333	Methadone
DB00334	Olanzapine
DB00335	Atenolol
DB00337	Pimecrolimus
DB00338	Omeprazole
DB00343	Diltiazem
DB00344	Protriptyline
DB00346	Alfuzosin
DB00349	Clobazam
DB00350	Minoxidil

DBID	Drug Name
DB00351	Megestrol
DB00356	Chlorzoxazone
DB00357	Aminoglutethimide
DB00358	Mefloquine
DB00360	Tetrahydrobiopterin
DB00361	Vinorelbine
DB00363	Clozapine
DB00364	Sucralfate
DB00367	Levonorgestrel
DB00368	Norepinephrine
DB00370	Mirtazapine
DB00371	Meprobamate
DB00373	Timolol
DB00374	Treprostinil
DB00376	Trihexyphenidyl
DB00377	Palonosetron
DB00379	Mexiletine
DB00380	Dexrazoxane
DB00381	Amlodipine
DB00382	Tacrine
DB00384	Triamterene
DB00388	Phenylephrine
DB00390	Digoxin
DB00393	Nimodipine
DB00396	Progesterone
DB00398	Sorafenib
DB00401	Nisoldipine
DB00404	Alprazolam
DB00408	Loxapine
DB00411	Carbachol
DB00412	Rosiglitazone
DB00413	Pramipexole
DB00418	Secobarbital
DB00419	Miglustat
DB00421	Spironolactone
DB00422	Methylphenidate
DB00423	Methocarbamol
DB00425	Zolpidem

DBID	Drug Name
DB00431	Lindane
DB00433	Prochlorperazine
DB00434	Cyproheptadine
DB00437	Allopurinol
DB00439	Cerivastatin
DB00440	Trimethoprim
DB00441	Gemcitabine
DB00444	Teniposide
DB00446	Chloramphenicol
DB00448	Lansoprazole
DB00449	Dipivefrin
DB00450	Droperidol
DB00454	Meperidine
DB00457	Prazosin
DB00458	Imipramine
DB00459	Acitretin
DB00461	Nabumetone
DB00462	Methylscopolamine
DB00465	Ketorolac
DB00471	Montelukast
DB00472	Fluoxetine
DB00474	Methohexital
DB00475	Chlordiazepoxide
DB00476	Duloxetine
DB00477	Chlorpromazine
DB00480	Lenalidomide
DB00481	Raloxifene
DB00482	Celecoxib
DB00484	Brimonidine
DB00486	Nabilone
DB00489	Sotalol
DB00490	Buspirone
DB00491	Miglitol
DB00492	Fosinopril
DB00494	Entacapone
DB00496	Darifenacin
DB00497	Oxycodone
DB00499	Flutamide

DBID	Drug Name
DB00500	Tolmetin
DB00501	Cimetidine
DB00502	Haloperidol
DB00518	Albendazole
DB00519	Trandolapril
DB00521	Carteolol
DB00530	Erlotinib
DB00532	Mephenytoin
DB00533	Rofecoxib
DB00535	Cefdinir
DB00537	Ciprofloxacin
DB00539	Toremifene
DB00540	Nortriptyline
DB00541	Vincristine
DB00542	Benazepril
DB00543	Amoxapine
DB00545	Pyridostigmine
DB00547	Desoximetasone
DB00548	Azelaic Acid
DB00549	Zafirlukast
DB00550	Propylthiouracil
DB00554	Piroxicam
DB00555	Lamotrigine
DB00558	Zanamivir
DB00559	Bosentan
DB00561	Doxapram
DB00563	Methotrexate
DB00564	Carbamazepine
DB00571	Propranolol
DB00572	Atropine
DB00573	Fenoprofen
DB00575	Clonidine
DB00580	Valdecoxib
DB00585	Nizatidine
DB00586	Diclofenac
DB00590	Doxazosin
DB00591	Fluocinolone Acetonide
DB00593	Ethosuximide

DBID	Drug Name
DB00594	Amiloride
DB00598	Labetalol
DB00602	Ivermectin
DB00603	Medroxyprogesterone
DB00605	Sulindac
DB00608	Chloroquine
DB00611	Butorphanol
DB00612	Bisoprolol
DB00615	Rifabutin
DB00619	Imatinib
DB00620	Triamcinolone
DB00621	Oxandrolone
DB00622	Nicardipine
DB00623	Fluphenazine
DB00624	Testosterone
DB00630	Alendronate
DB00631	Clofarabine
DB00633	Dexmedetomidine
DB00635	Prednisone
DB00640	Adenosine
DB00641	Simvastatin
DB00642	Pemetrexed
DB00647	Propoxyphene
DB00650	Leucovorin
DB00651	Dyphylline
DB00652	Pentazocine
DB00654	Latanoprost
DB00656	Trazodone
DB00659	Acamprosate
DB00661	Verapamil
DB00665	Nilutamide
DB00668	Epinephrine
DB00669	Sumatriptan
DB00672	Chlorpropamide
DB00673	Aprepitant
DB00674	Galantamine
DB00675	Tamoxifen
DB00678	Losartan

DBID	Drug Name
DB00679	Thioridazine
DB00680	Moricizine
DB00683	Midazolam
DB00685	Trovafloxacin
DB00687	Fludrocortisone
DB00690	Flurazepam
DB00691	Moexipril
DB00692	Phentolamine
DB00694	Daunorubicin
DB00695	Furosemide
DB00696	Ergotamine
DB00697	Tizanidine
DB00700	Eplerenone
DB00703	Methazolamide
DB00704	Naltrexone
DB00706	Tamsulosin
DB00708	Sufentanil
DB00710	Ibandronate
DB00712	Flurbiprofen
DB00714	Apomorphine
DB00715	Paroxetine
DB00720	Clodronate
DB00721	Procaine
DB00724	Imiquimod
DB00727	Nitroglycerin
DB00728	Rocuronium
DB00731	Nateglinide
DB00733	Pralidoxime
DB00734	Risperidone
DB00735	Naftifine
DB00740	Riluzole
DB00745	Modafinil
DB00747	Scopolamine
DB00749	Etodolac
DB00750	Prilocaine
DB00751	Epinastine
DB00753	Isoflurane
DB00754	Ethotoin

DBID	Drug Name
DB00757	Dolasetron
DB00758	Clopidogrel
DB00762	Irinotecan
DB00763	Methimazole
DB00764	Mometasone
DB00768	Olopatadine
DB00772	Malathion
DB00773	Etoposide
DB00774	Hydroflumethiazide
DB00775	Tirofiban
DB00776	Oxcarbazepine
DB00780	Phenelzine
DB00782	Propantheline
DB00783	Estradiol
DB00784	Mefenamic acid
DB00788	Naproxen
DB00790	Perindopril
DB00794	Primidone
DB00795	Sulfasalazine
DB00796	Candesartan
DB00798	Gentamicin
DB00799	Tazarotene
DB00800	Fenoldopam
DB00802	Alfentanil
DB00804	Dicyclomine
DB00806	Pentoxifylline
DB00807	Proparacaine
DB00808	Indapamide
DB00809	Tropicamide
DB00810	Biperiden
DB00811	Ribavirin
DB00813	Fentanyl
DB00814	Meloxicam
DB00818	Propofol
DB00819	Acetazolamide
DB00822	Disulfiram
DB00829	Diazepam
DB00831	Trifluoperazine

DBID	Drug Name
DB00834	Mifepristone
DB00835	Brompheniramine
DB00836	Loperamide
DB00838	Clocortolone
DB00839	Tolazamide
DB00841	Dobutamine
DB00842	Oxazepam
DB00843	Donepezil
DB00844	Nalbuphine
DB00850	Perphenazine
DB00851	Dacarbazine
DB00857	Terbinafine
DB00860	Prednisolone
DB00861	Diflunisal
DB00863	Ranitidine
DB00864	Tacrolimus
DB00868	Benzonatate
DB00869	Dorzolamide
DB00870	Suprofen
DB00871	Terbutaline
DB00872	Conivaptan
DB00873	Loteprednol
DB00876	Eprosartan
DB00881	Quinapril
DB00883	Isosorbide Dinitrate
DB00884	Risedronate
DB00887	Bumetanide
DB00889	Granisetron
DB00896	Rimexolone
DB00897	Triazolam
DB00898	Ethanol
DB00899	Remifentanil
DB00900	Didanosine
DB00903	Ethacrynic acid
DB00904	Ondansetron
DB00905	Bimatoprost
DB00906	Tiagabine
DB00907	Cocaine

DBID	Drug Name
DB00908	Quinidine
DB00909	Zonisamide
DB00910	Paricalcitol
DB00912	Repaglinide
DB00915	Amantadine
DB00918	Almotriptan
DB00920	Ketotifen
DB00921	Buprenorphine
DB00924	Cyclobenzaprine
DB00925	Phenoxybenzamine
DB00927	Famotidine
DB00929	Misoprostol
DB00933	Mesoridazine
DB00937	Diethylpropion
DB00938	Salmeterol
DB00949	Felbamate
DB00952	Naratriptan
DB00953	Rizatriptan
DB00959	Methylprednisolone
DB00960	Pindolol
DB00961	Mepivacaine
DB00962	Zaleplon
DB00963	Bromfenac
DB00964	Apraclonidine
DB00966	Telmisartan
DB00968	Methyldopa
DB00969	Alosetron
DB00973	Ezetimibe
DB00975	Dipyridamole
DB00978	Lomefloxacin
DB00979	Cyclopentolate
DB00980	Ramelteon
DB00981	Physostigmine
DB00983	Formoterol
DB00986	Glycopyrrolate
DB00988	Dopamine
DB00989	Rivastigmine
DB00990	Exemestane

DBID	Drug Name
DB00991	Oxaprozin
DB00992	Methyl aminolevulinate
DB00993	Azathioprine
DB00996	Gabapentin
DB00997	Doxorubicin
DB00998	Frovatriptan
DB00999	Hydrochlorothiazide
DB01001	Salbutamol
DB01005	Hydroxyurea
DB01006	Letrozole
DB01009	Ketoprofen
DB01012	Cinacalcet
DB01013	Clobetasol
DB01014	Balsalazide
DB01017	Minocycline
DB01018	Guanfacine
DB01019	Bethanechol
DB01023	Felodipine
DB01024	Mycophenolic acid
DB01029	Irbesartan
DB01030	Topotecan
DB01032	Probenecid
DB01035	Procainamide
DB01036	Tolterodine
DB01037	Selegiline
DB01039	Fenofibrate
DB01041	Thalidomide
DB01043	Memantine
DB01047	Fluocinonide
DB01050	Ibuprofen
DB01057	Echothiophate
DB01059	Norfloxacin
DB01062	Oxybutynin
DB01064	Isoproterenol
DB01067	Glipizide
DB01068	Clonazepam
DB01069	Promethazine
DB01073	Fludarabine

DBID	Drug Name
DB01076	Atorvastatin
DB01079	Tegaserod
DB01083	Orlistat
DB01085	Pilocarpine
DB01086	Benzocaine
DB01087	Primaquine
DB01088	Iloprost
DB01091	Butenafine
DB01095	Fluvastatin
DB01097	Leflunomide
DB01098	Rosuvastatin
DB01100	Pimozide
DB01101	Capecitabine
DB01104	Sertraline
DB01105	Sibutramine
DB01106	Levocabastine
DB01109	Heparin
DB01110	Miconazole
DB01114	Chlorpheniramine
DB01115	Nifedipine
DB01118	Amiodarone
DB01119	Diazoxide
DB01120	Gliclazide
DB01122	Ambenonium
DB01126	Dutasteride
DB01128	Bicalutamide
DB01129	Rabeprazole
DB01130	Prednicarbate
DB01132	Pioglitazone
DB01133	Tiludronate
DB01136	Carvedilol
DB01142	Doxepin
DB01143	Amifostine
DB01148	Flavoxate
DB01149	Nefazodone
DB01151	Desipramine
DB01156	Bupropion
DB01157	Trimetrexate

DBID	Drug Name
DB01158	Bretylium
DB01159	Halothane
DB01161	Chloroprocaine
DB01162	Terazosin
DB01165	Ofloxacin
DB01167	Itraconazole
DB01169	Arsenic trioxide
DB01173	Orphenadrine
DB01174	Phenobarbital
DB01177	Idarubicin
DB01182	Propafenone
DB01183	Naloxone
DB01184	Domperidone
DB01185	Fluoxymesterone
DB01186	Pergolide
DB01189	Desflurane
DB01193	Acebutolol
DB01194	Brinzolamide
DB01195	Flecainide
DB01196	Estramustine
DB01197	Captopril
DB01198	Zopiclone
DB01200	Bromocriptine
DB01202	Levetiracetam
DB01203	Nadolol
DB01204	Mitoxantrone
DB01205	Flumazenil
DB01206	Lomustine
DB01210	Levobunolol
DB01214	Metipranolol
DB01215	Estazolam
DB01216	Finasteride
DB01217	Anastrozole
DB01218	Halofantrine
DB01219	Dantrolene
DB01220	Rifaximin
DB01221	Ketamine
DB01222	Budesonide

DBID	Drug Name
DB01223	Aminophylline
DB01224	Quetiapine
DB01226	Mivacurium
DB01229	Paclitaxel
DB01233	Metoclopramide
DB01234	Dexamethasone
DB01236	Sevoflurane
DB01238	Aripiprazole
DB01241	Gemfibrozil
DB01242	Clomipramine
DB01247	Isocarboxazid
DB01248	Docetaxel
DB01250	Olsalazine
DB01254	Dasatinib
DB01258	Aliskiren
DB01260	Desonide
DB01261	Sitagliptin
DB01267	Paliperidone
DB01268	Sunitinib
DB01273	Varenicline
DB01275	Hydralazine
DB01276	Exenatide
DB01278	Pramlintide
DB01280	Nelarabine
DB01291	Pirbuterol
DB01306	Insulin Aspart
DB01320	Fosphenytoin
DB01327	Cefazolin
DB01337	Pancuronium
DB01340	Cilazapril
DB01356	Lithium
DB01364	Ephedrine
DB01367	Rasagiline
DB01373	Calcium
DB01378	Magnesium
DB01393	Bezafibrate
DB01394	Colchicine
DB01399	Salsalate

DBID	Drug Name	DBID	Drug Name	DBID	Drug Name
DB01400	Neostigmine	***DB01621***	Pipotiazine	***DB06209***	Prasugrel
DB01406	Danazol	***DB01623***	Thiothixene	***DB06228***	Rivaroxaban
DB01409	Tiotropium	***DB02300***	Calcipotriol	***DB06274***	Alvimopan
DB01410	Ciclesonide	***DB04835***	Maraviroc	***DB06287***	Temsirolimus
DB01427	Amrinone	***DB04839***	Cyproterone	***DB06335***	Saxagliptin
DB01558	Bromazepam	***DB04844***	Tetrabenazine	***DB06695***	Dabigatran etexilate
DB01577	Methamphetamine	***DB04845***	Ixabepilone	***DB06698***	Betahistine
DB01586	Ursodeoxycholic acid	***DB04861***	Nebivolol	***DB06699***	Degarelix
DB01591	Solifenacin	***DB04868***	Nilotinib	***DB06700***	Desvenlafaxine
DB01595	Nitrazepam	***DB04896***	Milnacipran	***DB06702***	Fesoterodine
DB01611	Hydroxychloroquine	***DB04930***	Permethrin	***DB06710***	Methyltestosterone
DB01612	Amyl Nitrite	***DB05246***	Methsuximide	***DB06711***	Naphazoline
DB01618	Molindone	***DB05271***	Rotigotine	***DB06802***	Nepafenac

Drugs were obtained from the DrugBank database[2], and their side effects were collected from the SIDER database[3].

Table 2 | The keywords used in the filtering of the DrugBank database and their occurrences

Keyword	Mark	Occurrences
„cancer”/ „lymphoma”/ „carcinoma”/ „leukemia”/ „tumor”	Anti-cancer	172
„colon”/ „colorectal”/ „carcinoma”/ „cancer”/ „tumor”	Anti-colorectal cancer	11
„diabetes mellitus”	Anti-diabetes	36

The keywords are listed which were used in the filtering of the DrugBank database[2] and their occurrences is noted. The plus sign (+) represents the “AND” logical operator, the slash (/) represents the “OR” logical operator.

Table 3 | Drugs obtained from the DrugBank database, which are used in the treatment of colorectal cancer and have no reported side effects in the SIDER database and their target proteins

DrugBank ID	Drug Name	Drug Target Proteins
DB00002	Cetuximab	O75015, P00533, P00736, P02745, P02746, P02747, P09871, P12314, P12318, P31994
DB00112	Bevacizumab	O75015, P00736, P02745, P02746, P02747, P12314, P12318, P31994
DB00113	Arcitumomab	P13688
DB00544	Fluorouracil	P04818
DB00848	Levamisole	P10696, P32297
DB01269	Panitumumab	P00533
DB01873	Epothilone D	P04350, P07437, P68363, P68366, P68371, Q13509, Q13748, Q71U36, Q9BQE3, Q9H4B7, Q9NY65

Drugs and their targets were obtained from the DrugBank database[2]. Only those drugs were selected, which are used in the treatment of colorectal cancer and have no reported side effects in the SIDER database[3]. Target proteins for each drug were identified by their UniProt ID[9].

Table 4 │ Drugs obtained from the DrugBank database, which are used in the treatment of colorectal cancer and have known side effects in the SIDER database and their target proteins

Drugbank ID	Drug Name	Drug Target Proteins
DB00650	Leucovorin	P04818
DB00762	Irinotecan	P11387
DB01101	Capecitabine	P04818
DB01157	Trimetrexate	P00374

Drugs and their targets were obtained from the DrugBank database[2]. Only those drugs were selected, which are used in the treatment of colorectal cancer and have known side effects in the SIDER database[3]. Target proteins for each drug were identified by their UniProt ID[9].

Table 5 | Drugs obtained from the DrugBank database, which are used in the treatment of type 2 diabetes and have no reported side effects in the SIDER database and their target proteins

DrugBank ID	Drug Name	Drug Target Proteins
DB00030	Insulin recombinant	P06213, P06400, P07339, P08069, P14735, P16519, P16870, P29120, P48745, P98164, Q16270, Q96C24
DB00071	Insulin, porcine	P01906, P06213, P06400, P07339, P08069, P14735, P16519, P16870, P29120, P48745, P98164, Q16270, Q96C24
DB00414	Acetohexamide	P48048
DB00722	Lisinopril	P12821, Q9BYF1
DB00914	Phenformin	Q13131, Q15842
DB01124	Tolbutamide	P48048, Q09428
DB01251	Gliquidone	Q09428, Q15842
DB01289	Glisoxepide	Q09428, Q15842
DB01307	Insulin Detemir	P06213
DB01309	Insulin Glulisine	P06213
DB01382	Glycodiazine	P48048, Q09428
DB04876	Vildagliptin	P27487
DB06655	Liraglutide	P43220

Drugs and their targets were obtained from the DrugBank database[2]. Only those drugs were selected, which are used in the treatment of type 2 diabetes and have no reported side effects in the SIDER database[3]. Target proteins for each drug were identified by their UniProt ID[9].

Table 6 | Drugs obtained from the DrugBank database, which are used in the treatment of type 2 diabetes and have known side effects in the SIDER database and their target proteins

Drugbank ID	Drug Name	Drug Target Proteins
DB00046	Insulin Lispro	P06213, P08069
DB00047	Insulin Glargine	P06213, P08069
DB00178	Ramipril	P12821
DB00197	Troglitazone	O60488, P05121, P11474, P37231, P62508, Q99808
DB00222	Glimepiride	P48048, Q09428, Q14654
DB00412	Rosiglitazone	O60488, P37231
DB00491	Miglitol	P10253, Q14697, Q8TET4
DB00492	Fosinopril	P12821
DB00519	Trandolapril	P12821
DB00731	Nateglinide	P37231, Q09428
DB00834	Mifepristone	P04150, P06401
DB00839	Tolazamide	P48048
DB00881	Quinapril	P12821
DB00912	Repaglinide	P37231, Q09428
DB00966	Telmisartan	P30556, P37231
DB01067	Glipizide	P37231, Q09428
DB01132	Pioglitazone	P37231
DB01261	Sitagliptin	P27487
DB01276	Exenatide	P43220
DB01278	Pramlintide	O60894, O60895, O60896
DB01306	Insulin Aspart	P06213
DB01393	Bezafibrate	P37231, Q03181, Q07869
DB06335	Saxagliptin	P27487

Drugs and their targets were obtained from the DrugBank database[2]. Only those drugs were selected, which are used in the treatment of type 2 diabetes and have known side effects in the SIDER database[3]. Target proteins for each drug were identified by their UniProt ID[9].

Table 7 | Mutated genes in colorectal cancer and their corresponding proteins

Gene name	Protein identifier
AKT1	P31749
APC	P25054
BRAF	P15056
CTNNB1	P35222
EP300	Q09472
FBXW7	Q969H0
KRAS	P01116
MADH4	Q13485
MAP2K4	P45985
MDM2	Q00987
MLH1	P40692
MSH2	P43246
MSH6	P52701
PIK3CA	P42336
PIK3R1	P27986
TCF7L2	Q9NQB0
TP53	P04637
VTI1A	Q96AJ9

The 18 mutated genes in colorectal cancer were obtained from the Cancer Gene Census[10] and the proteins coded by them were mapped by PICR[11].

Table 8 | Mutated genes in type 2 diabetes and their corresponding proteins

Gene name	Protein identifier	Gene name	Protein identifier
ABCC8	Q54P13	***KCNQ1***	P51787*
CAPN10	Q9HC96	***IRS1***	Q28224
HNF1B	Q91910	***MTNR1B***	Q8CIQ6
GCGR	P30082	***PROX1***	P48437
TCF7L2	Q9NQB0*	***GCKR***	Q07071
PPARG	O18924	***ADCY5***	P30803
KCNJ11	O02822	***UBE2E2***	Q96LR5*
WFS1	P56695	***BCL11A***	Q9H165*
HNF1B	Q91910	***GCKR***	Q07071
SLC30A8	Q5I020	***DGKB***	Q9Y6T7*
HHEX	D2KQB0	***TMEM195***	A0JPQ8
CDKAL1	Q5VV42*	***C2CD4B***	A6NLJ0
IGF2BP2	Q9Y6M1*	***KLF14***	Q9ESX2
CDKN2A	O77617	***ZBED3***	Q96IU2
CDKN2B	P42772*	***TP53INP1***	Q96A56*
FTO	Q9C0B1*	***CHCHD9***	Q5T1J5
JAZF1	Q80ZQ5	***CENTD2***	Q4LDD4
CDC123	A6R687	***HMGA2***	P52926*
CAMK1D	Q8IU85*	***HNF1A***	Q90867
TSPAN8	Q2KIS9	***PRC1***	Q94JQ6
LGR5	Q9Z1P4	***ZFAND6***	Q9DCH6
THADA	A8C752	***DUSP9***	Q99956*
ADAMTS9	Q9P2N4		
NOTCH2	Q04721*		

The 46 mutated genes in type 2 diabetes were obtained from the article of Parchwani et al.[12] and the proteins coded by them were mapped by PICR[10]. From the 46 proteins listed here only 14 were contained in the human interactome constructed from the STRING database[1]; those are marked with an asterisk (*) in the Table.

Table 9 | Average human interactome centralities of target proteins of drugs against colorectal cancer and type 2 diabetes

Centrality type	Drug targets without side effects			Drug targets with side effects		
	Colorectal cancer	Type 2 diabetes	Statistical difference	Colorectal cancer	Type 2 diabetes	Statistical difference
**Degree** (number of neighbours)	24.50	13.00	0.203	40.00	34.00	0.941
**Closeness centrality** (1/edge)	0.305	0.295	0.330	0.301	0.292	0.572
**Betweenness centrality** (fraction of shortest paths passing through the node)	1.46E-4	5.76E-4	0.601	3.39E-4	1.28E-4	0.944

The table shows the medians of the centralities of target proteins of drugs against colorectal cancer and type 2 diabetes without or with reported side effects (the results were very similar, if instead of medians we used the arithmetic means; data not shown). Centrality values were calculated with the Pajek programme[6]. The human interactome containing 12,439 proteins and 174,666 edges was built from the STRING database[1], 1,726 human drug targets were obtained from the DrugBank database[2], and the proteins were labelled by their UniProt ID[9]. 99,423 drug-side effect pairs were taken from the SIDER database[3]. Statistical analysis was performed using the Wilcoxon rank sum (Mann-Whitney) test function of the R package[5].

Table 10 | Average network distance between drug targets without known side effects used in the treatment of colorectal cancer and colorectal cancer-associated proteins

**UniProt ID of colorectal cancer drug targets without side effects**	**Average network distance from colorectal cancer-related proteins** (edges)
O75015	2.500
P00533	1.722
P00736	2.722
P02745	2.889
P02746	3.000
P02747	3.000
P04350	2.278
P07437	2.167
P09871	3.000
P10696	3.222
P12314	2.722
P12318	2.444
P13688	2.444
P31994	2.500
P32297	3.056
P68363	2.111
P68366	2.000
P68371	2.444
Q13509	2.722
Q13748	2.111
Q71U36	2.111
Q9BQE3	2.389
Q9H4B7	2.778
Q9NY65	2.333
Mean network distance of drug targets	**2.528**
Mean network distance of randomly selected proteins	**3.316**

The table shows the average network distance between drug targets without known side effects used in the treatment of colorectal cancer and colorectal cancer-related proteins. The total number of drug targets without known side effects used in the treatment of colorectal cancer was 24; the total number of colorectal cancer-related proteins was 18. Average network distances were calculated as shortest paths using the Pajek programme[6]. Proteins were labelled by their UniProt ID[9]. The human interactome containing 12,439 proteins and 174,666 edges was built from the STRING database[1], 1,726 human drug targets were obtained from the DrugBank database[2] and 99,423 drug-side effect pairs were taken from the SIDER database[3]. Colorectal cancer-related proteins were obtained from the Cancer Gene Census database[10]. Average network distances between colorectal cancer-related proteins and at least 50 randomly selected samples of 24 proteins each were calculated, and the statistical difference in their mean values compared to the average network distance of the 24 drug targets listed above was tested using the one-way ANOVA (Analysis of Variance) with linear model fit function of the R package[5]. There was no statistically significant difference between the mean values of the drug targets without known side effects and the random samples, $F=0.8807$, $p=0.7078$.

Table 11 | Average network distance between drug targets with known side effects used in the treatment of colorectal cancer and colorectal cancer-associated proteins

**UniProt ID of colorectal cancer drug targets with side effects**	**Average network distance from colorectal cancer-related proteins** (edges)
P00374	2.500
P04818	2.556
P11387	2.111
Mean network distance of drug targets	**2.389**
Mean network distance of randomly selected proteins	**3.240**

The table shows the average network distance between drug targets with known side effects used in the treatment of colorectal cancer and colorectal cancer-related proteins. The total number of drug targets with known side effects used in the treatment of colorectal cancer was 3; the total number of colorectal cancer-related proteins was 18. Average network distances were calculated as shortest paths using the Pajek programme[6]. Proteins were labelled by their UniProt ID[9]. The human interactome containing 12,439 proteins and 174,666 edges was built from the STRING database[1], 1,726 human drug targets were obtained from the DrugBank database[2] and 99,423 drug-side effect pairs were taken from the SIDER database[3]. Colorectal cancer-related proteins were obtained from the Cancer Gene Census database[10]. Average network distances between colorectal cancer related proteins and at least 50 randomly selected samples of 3 proteins each were calculated, and the statistical difference in their mean values compared to the average network distance of the 3 drug targets listed above was tested using the one-way ANOVA (Analysis of Variance) with linear model fit function of the R package[5]. There was no statistically significant difference between the mean values of the drug targets with known side effects and the random samples, $F=1.223$, $p=0.1951$.

Table 12 | Average network distance between drug targets without known side effects used in the treatment of type 2 diabetes and diabetes-associated proteins

**UniProt ID of type 2 diabetes drug targets without side effects**	**Average network distance from diabetes-related proteins** (edges)
P01906	*3.786*
P06400	*2.286*
P07339	*3.000*
P14735	*3.214*
P16519	*3.786*
P16870	*3.286*
P29120	*3.143*
P48745	*3.214*
P98164	*3.000*
Q13131	*2.929*
Q15842	*3.714*
Q16270	*3.143*
Q96C24	*3.500*
Q9BYF1	*3.500*
Mean network distance of drug targets	***3.250***
Mean network distance of randomly selected proteins	***3.413***

The table shows the average network distance between drug targets without known side effects used in the treatment of type 2 diabetes and diabetes-related proteins. The total number of drug targets without known side effects used in the treatment of type 2 diabetes was 14; the total number of type 2 diabetes-related proteins contained in the human interactome was 14. Average network distances were calculated as shortest paths using the Pajek programme[6]. Proteins were labelled by their UniProt ID[9]. The human interactome containing 12,439 proteins and 174,666 edges was built from the STRING database[1], 1,726 human drug targets were obtained from the DrugBank database[2] and 99,423 drug-side effect pairs were taken from the SIDER database[3]. Type 2 diabetes-related proteins were obtained from the article of Parchwani et al.[12]. Average network distances between type-2 diabetes related proteins and at least 50 randomly selected samples of 14 proteins each were calculated, and the statistical difference in their mean values compared to the average network distance of the 14 drug targets listed above was tested using the one-way ANOVA (Analysis of Variance) with linear model fit function of the R package[5]. There was no statistically significant difference between the mean values of the drug targets without known side effects and the random samples, $F=0.7867$, $p=0.8547$.

Table 13 | Average network distance between drug targets with known side effects used in the treatment of type 2 diabetes and diabetes-associated proteins

**UniProt ID of type 2 diabetes drug targets with side effects**	**Average network distance from diabetes-related proteins** (edges)
O60488	3.643
O60894	3.857
O60895	3.857
O60896	3.429
P04150	2.429
P05121	2.857
P06213	2.643
P06401	2.500
P08069	2.571
P10253	3.786
P11474	3.000
P12821	3.786
P27487	3.500
P30556	3.000
P37231	2.643
P43220	3.071
P48048	3.214
P62508	3.071
Q03181	2.857
Q07869	2.714
Q09428	3.500
Q14654	3.286
Q14697	3.357
Q8TET4	3.929
Q99808	4.357
Mean network distance of drug targets	**3.234**
Mean network distance of randomly selected proteins	**3.443**

The table shows the average network distance between drug targets with known side effects used in the treatment of type 2 diabetes and diabetes-related proteins. The total number of drug targets with known side effects used in the treatment of type 2 diabetes was 25; the total number of type 2 diabetes-related proteins contained in the human interactome was 14. Average network distances were calculated as shortest paths using the Pajek programme[6]. Proteins were labelled by their UniProt ID[9]. The human interactome containing 12,439 proteins and 174,666 edges was built from the STRING database[1], 1,726 human drug targets were obtained from the DrugBank database[2] and 99,423 drug-side effect pairs were taken from the SIDER database[3]. Type 2 diabetes-related proteins were obtained from the article of Parchwani et al.[12]. Average network distances between type-2 diabetes related proteins and at least 50 randomly selected samples of 25 proteins each were calculated, and the statistical difference in their mean values compared to the average network distance of the 25 drug targets listed above was tested using the one-way ANOVA (Analysis of Variance) with linear model fit function of the R package[5]. There was no statistically significant difference between the mean values of the drug targets with known side effects and the random samples, $F= 0.9021$, $p= 0.6677$.

Supplementary References

1. Franceschini, A. *et al.* STRING v9.1: protein-protein interaction networks, with increased coverage and integration. *Nucleic Acids Res.* **41,** D808–D815 (2012).
2. Knox, C. *et al.* DrugBank 3.0: a comprehensive resource for 'omics' research on drugs. *Nucleic Acids Res.* **39,** D1035–1041 (2011).
3. Kuhn, M., Campillos, M., Letunic, I., Jensen, L. J. & Bork, P. A side effect resource to capture phenotypic effects of drugs. *Mol. Syst. Biol.* **6,** 343 (2010).
4. Szalay, K. Z. & Csermely, P. Perturbation centrality and Turbine: A novel centrality measure obtained using a versatile network dynamics tool. *PLoS ONE* **8,** e78059 (2013).
5. R Core Team. *R: A language and environment for statistical computing.* (R Foundation for Statistical Computing, 2013). at <http://www.R-project.org/>
6. Vladimir Bagatelj & Andrej Mrvar. in *Graph drawing software* (eds. Jünger, M. & Mutzel, P.) 77–103 (Springer, 2003). at <http://pajek.imfm.si/>
7. The Inkscape Team. *Inkscape*. (2014). at <http://inkscape.org>
8. Shannon, P. *et al.* Cytoscape: a software environment for integrated models of biomolecular interaction networks. *Genome Res.* **13,** 2498–2504 (2003).
9. The UniProt Consortium. Reorganizing the protein space at the Universal Protein Resource (UniProt). *Nucleic Acids Res.* **40,** D71–D75 (2012).
10. Forbes, S. A. *et al.* COSMIC: mining complete cancer genomes in the Catalogue of Somatic Mutations in Cancer. *Nucleic Acids Res.* **39,** D945–D950 (2010).
11. Wein, S. P. *et al.* Improvements in the Protein Identifier Cross-Reference service. *Nucleic Acids Res.* **40,** W276–280 (2012).
12. Parchwani, D., Murthy, S., Upadhyah, A. & Patel, D. Genetic factors in the etiology of type 2 diabetes: linkage analyses, candidate gene association, and genome-wide association – still a long way to go! *Natl. J. Physiol. Pharm. Pharmacol.* **3,** 57 (2013).
